# Response to Tsikas et al. Comments on Boelaert et al. Determination of Asymmetric and Symmetric Dimethylarginine in Serum from Patients with Chronic Kidney Disease: UPLC–MS/MS versus ELISA. *Toxins* 2016, *8*, 149

**DOI:** 10.3390/toxins8110312

**Published:** 2016-10-27

**Authors:** Jente Boelaert, Eva Schepers, Griet Glorieux, Sunny Eloot, Raymond Vanholder, Frédéric Lynen

**Affiliations:** 1 Department of Organic Chemistry, Separation Science Group, Ghent University, Krijgslaan 281, S4-bis, B-9000 Ghent, Belgium; jente.boelaert@gmail.com; 2 Department of Internal Medicine, Nephrology Section, Ghent University Hospital, De Pintelaan 185, B-9000 Ghent, Belgium; eva.schepers@ugent.be (E.S.); griet.glorieux@ugent.be (G.G.); sunny.eloot@ugent.be (S.E.); raymond.vanholder@ugent.be (R.V.)

Tsikas et al. question the validity of both the UPLC–MS/MS and ELISA method, a possible cause of the observed discrepancies in the asymmetric dimethylarginine (ADMA) and symmetric dimethylarginine (SDMA) quantifications observed in the paper of Boelaert et al. [1,2]. It is of note that LC–MS/MS is probably the most accurate technique developed thus far for biomolecule analysis in complex samples. Although any analytical methodology should always be scrutinized for possible weaknesses, the proposed UPLC–MS/MS methodology has been designed for robustness and reliability, and underwent a rigid validation protocol to anticipate possible methodological weaknesses.

In addition, we find it worthwhile to stress some other measures taken by our group to guarantee the reliability of the UPLC–MS/MS method:

1. In contrast to the earlier cited HPLC–MS/MS article by Schwedhelm et al. [3] the UPLC–MS/MS approach with a 1.7 µM particle column allows for complete resolution of ADMA and SDMA on the time scale axis of the chromatogram. Therefore, spectral resolution proposed in 2005 is essentially redundant in the UPLC–MS/MS methodology. Our group always tries to combine full chromatographic with spectral resolution to anticipate on co-elution issues which might complicate MS ionization and fragmentation, as this makes methods more robust. Thus the use of MS/MS fragmentation data in combination with baseline chromatographic separations allows for the most selective detection possible today [4,5]. The improved separation, the higher column efficiency and the higher solute retention also makes the method less sensitive to matrix effects, typically eluting close to the void time [6] in comparison to earlier approaches [3].
2. Throughout our work, a deuterated internal standard was used to correct for all possible variations in extraction efficiency and possible losses prior to chromatography and for possible, but unlikely, discrepancies at injection or during chromatography. The use of a deuterated internal standard is in accordance with the best practices in LC–MS/MS and becomes especially important when reaching the lower ppb level (0.1–10 ppb) limits, because at those levels matrix effects due to, for example, phospholipids might become relevant and can perturb quantitative analyses [6,7,8]. As the lowest measured ADMA/SDMA concentrations in serum in our study were in the 50–100 ppb range, the influence of the matrix affecting ionization efficiency seems unlikely.
3. The quality of the UPLC–MS/MS method was checked by us with quality control (QC) samples at the lower, middle and high end of the calibration curve. A maximal deviation of 12.35% was measured in this way for SDMA for the lowest point. We agree that higher QC accuracy is preferable but these deviations are too low to explain the differences between the UPLC–MS/MS and the ELISA data.
4. As the limit of detection (LOD) and limit of quantification (LOQ) concentration limits of the method are much smaller than the actual onset concentrations of ADMA and SDMA in serum (6.4 and 7.9 nM versus ~500 nM) false positive identifications can be excluded.

Tsikas et al. state that the larger deviations between UHPLC–MS/MS and ELISA in the middle of the concentration range could be related to varying fragmentation patterns of SDMA, ADMA and to the use of only d7-ADMA in the MS [1]. Firstly, all samples, including serum, calibrating samples, and internal standards were esterified with butanol and further treated in exactly the same way. Furthermore, the degree of fragmentation in LC–MS/MS is concentration independent since in MS each molecule behaves independently and at the applied vacuum levels should have a mean free path of about 0.5–1 m. If fragmentation in the MS/MS collision cell were concentration dependent, the technique would not be able to generate linear calibration curves (within the electrospray ionization (ESI) reachable zone of about two orders of magnitude) and would not be used as it is today for quantitative analysis in life sciences. We agree that each molecule, even deuterated standards, will fragment somewhat differently, however the fragmentation pattern must be (and is) constant in MS/MS. The fact that an internal standard fragments somewhat differently compared to the analytes of interest is not atypical and can cause no problem as the ratio between the eventually measured daughter ions will always be constant. The main purpose of the internal standard is to correct for extraction, sample preparation and possibly (at lower ppb levels) for ionization efficiency variations. Once the ions enter the high vacuum zone they behave independently of each other and the utility of the internal standard becomes reduced as the ratios of fragmentation are constant.

The referred work of Schwedhelm et al. [3] is highly interesting, and discusses interesting discrepancies between LC–MS and LC–MS/MS, and suggests that fragmentation patterns of the butylester derivatives could be at the origin of the measured discrepancies between UPLC–MS/MS and ELISA. As an alternative, it might be hypothesized that the discrepancies are related to the ELISAs, as also found by others [5,9].

However, as stressed by Tsikas et al. the matrix used for calibration might be a confounder. In this context we recently quantified the concentration for a range of compounds with a variable degree of protein binding, applying both a standard curve prepared in an aqueous buffer and in serum. The concentrations obtained with the calibration curve in serum were 1% to 21% lower than those calculated using the curve prepared in aqueous buffer (unpublished data). So we agree that dissolving calibrators in a matrix equal to the samples, being serum, is preferable. Nevertheless, to minimize background signals, serum should be cleared from internal metabolites, for example, by using active carbon as described by Itoh et al. [10].

In addition, we agree that for determination of protein binding of solutes, HSA-containing phosphate buffer can be considered as the ideal matrix. However, serum is the in vivo relevant matrix and, in the context of CKD, the competition for protein binding by the many retained uremic solutes and drugs taken by those patients also needs to be considered [11], whereas those are absent in the buffer recommended above. Therefore serum might better reflect real-life conditions.

Another issue raised by Tsikas et al. addresses the statistical comparison between both the UPLC–MS/MS and the ELISA method. Although we acknowledge that in Figure 3 of our paper [2], the dots on the correlation plots diverge from the identity line, the mutual relation between ELISA and UPLC–MS/MS was demonstrated to be linear for both ADMA and SDMA, be it with rather small but significant correlation coefficients (*R* = 0.78 for ADMA; *R* = 0.72 for SDMA; *p* < 0.0001). Since for ADMA the regression line is parallel to the identity line, ELISA seems to overestimate ADMA concentrations. For SDMA, the deviation of the regression line from the identity line points to the fact that the assumption of analytical linearity, in one of the methods, is incorrect. While for the UPLC–MS/MS method, linearity was demonstrated (*r*² > 0.99), the ELISA method might lack linearity, causing disagreement. It is of note that the ELISA standard curve for SDMA only covers a concentration range from 0 to 3.0 µM (for which the supplier guarantees linearity (98% (90%–106%)) and the second highest standard contains 0.8 µM SDMA. However, in CKD5HD patients’ SDMA concentration is mostly higher than 0.8 µM and often above the upper limit of 3 µM. In the specific patient population presented in the paper by Boelaert et al. 95% had a concentration above 0.8 µM, 14% of which had a concentration higher than 3.0 µM [2]. This limited concentration range for SDMA in ELISA might therefore contribute to the disagreement between ELISA and UPLC–MS/MS, again suggesting that the discrepancy in the results might be more attributed to shortcomings of the ELISA methods rather than the UPLC–MS/MS method.

Finally, for evaluating the degree of protein binding of ADMA and SDMA, the samples were prepared as described in paragraph 4.2.3 of our paper, but it should be underlined that both deproteinized and non-deproteinized samples for determination of free fraction underwent the same ultrafiltration treatment (ultrafiltration using Millipore Centrifree devices (MWCO 30,000 Da) at 1469× *g* for 25 min). 

This method has been used extensively in our laboratory for a long time and enables us to quantify the degree of protein binding of a panel of uremic retention solutes with a variable degree of protein binding. For example, as described by Deltombe et al. where the same ultrafiltration method was used (1469× *g* for 25 min), this approach resulted in the finding of a protein binding for hemodialysis (HD) patients of 95% (93; 97) for *p*-cresylsulphate and 39% (32; 54) for hippuric acid [12], corresponding well to protein binding measured by Itoh et al. of 95.1% ± 0.6% and 48.3% ± 2.5% respectively, who used ultracentrifugation (20,600× *g*, 10 min) [10] and to Viaene et al. who found a protein binding of 91.9% for *p*-cresylsulphate with a similar ultrafiltration (2000× *g*, 90 min) [13]. It is of note that, in our method, centrifugation forces (1469× *g*, 25 min) are five times higher than the ones used by Sitar et al. (300× *g*, 1 min) [14], and performed for a longer period of time. We do agree with Tsikas et al. that protein binding and the kinetic pattern of ADMA and SDMA in CKD is a complex process, and that further mechanistic studies are warranted [1].
